# Hepatorenal Toxicity after 7-Day Oral Administration of Low-Dose Tetrodotoxin and Its Distribution in the Main Tissues in Mice

**DOI:** 10.3390/toxins15090564

**Published:** 2023-09-08

**Authors:** Yaqian Zhong, Xiaojun Zhang, Qiyu Yang, Qianfeng Wang

**Affiliations:** 1 School of Food and Pharmacy, Zhejiang Ocean University, Zhoushan 316022, China; zhongyaqian@zjou.edu.cn (Y.Z.); yangqiyu@zjou.edu.cn (Q.Y.); wangqianfeng@zjou.edu.cn (Q.W.); 2 Laboratory of Aquatic Product Processing and Quality Safety, Zhejiang Marine Fisheries Research Institute, Zhoushan 316100, China

**Keywords:** tetrodotoxin, mice, hepatorenal toxicity, low dose

## Abstract

Tetrodotoxin (TTX) is a highly toxic compound detected in various edible marine animals even in European waters. To characterize the hazard by oral exposure to TTX, its tissue distribution was evaluated after single (75 μg/kg) or 7-day (25–125 μg/kg) oral administration in mice. Moreover, TTX liver and renal toxicity was evaluated after 7-day oral administration. The elimination cycle of a single oral dose of TTX (75 µg/kg) was found to be approximately 168 h (7 days). Daily oral administration of TTX at doses of 25, 75, and 125 µg/kg for 7 consecutive days revealed dose-dependent toxic effects on the liver and kidney. Histopathological examination showed increased inflammatory cell infiltration in the liver and kidney with higher TTX doses, along with disorganization of the hepatic cord and renal tubular cell arrangement. The study results indicated that TTX had more hepatotoxicity than nephrotoxicity in mice. These findings provide insights into the unintentional ingestion of a low dose of TTX in mammals, including humans, and emphasize the importance of food safety.

## 1. Introduction

Tetrodotoxin (TTX) is a highly toxic marine neurotoxin [1] which was originally found mainly in pufferfish, and so was named tetrodotoxin [2]. Tetrodotoxin can selectively bind to voltage-dependent sodium (Nav) conductance channels in nerve and muscle cells, blocking the influx of sodium ions through the channel and thus preventing the generation and propagation of action potentials [3]. TTX plays a role in the processes analgesia, anesthesia, and detoxification processes, so it is often used as a blocking agent in medical research [4,5]. The incubation period of TTX poisoning is short and there is no specific antidote. The main poisoning symptoms are paralysis of the central and peripheral nervous system, abdominal pain and diarrhea, respiratory failure, and even respiratory arrest in severe cases [6]. TTX poisoning is mainly caused by human consumption of seafood containing or contaminated with TTX, which mostly occurs in China, Japan, and other Asian countries [7]. In recent years, TTX has been identified in fish and bivalve mollusks caught in European waters, leading to increased concern about the risks of TTX to public health [8,9,10,11].

Mice have a low resistance to TTX [12]. It has been described that, in mice, the acute oral lethal dose (LD_50_) of TTX is 232 μg/kg BW, and the acute oral No Observed Adverse Effect Level (NOAEL) is 75 μg/kg BW [13]. Recently, several research groups have attempted to comprehend the possible mechanisms of TTX accumulation and metabolism from various perspectives. in order to prevent the occurrence of TTX poisoning [14,15,16,17]. TTX is at increased risk of accidental ingestion, but studies on the effects of sustained ingestion of a low dose of TTX in humans are still scarce. Given the high number of acute toxicity studies on TTX [18,19,20], people are better prepared for acute toxicity of TTX, so the European Food Safety Authority (EFSA) has emphasized the need for chronic toxicity studies on TTX. The short-term accumulation distribution and metabolism of TTX in mammals and the extent of damage to body tissues are not known.

The purpose of this study was to investigate the tissue distribution of TTX in mice following single oral and daily oral administration of TTX, as well as the toxic effects on the liver and kidneys caused by oral administration of tetrodotoxin to mice for 7 days. For this purpose, in single-dose administration experiments, mice were administered by gavage and the levels of TTX in different tissues of mice were assessed. In daily oral administration experiments, TTX levels in liver and kidney, oxidative stress indices, liver and kidney function indices in serum, and histopathological examination of liver and kidney were quantified in mice. This provides a reference for unintentional ingestion of a low dose of tetrodotoxin in mammals as well as humans.

## 2. Results

### 2.1. Single Oral Administration of TTX in Mice: Temporal Profile of Its Distribution in Blood and Selected Tissues

**Figure 1:**
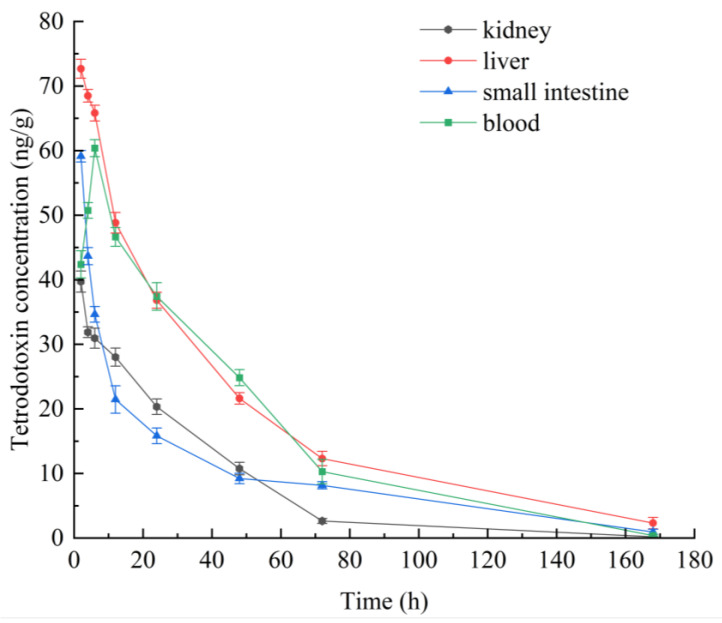
Temporal profile of TTX concentration in the blood, kidney, liver, and small intestine of mice. The data are from a single oral gavage administered to mice at a dose of 75 μg/kg TTX. Data represent mean ± SE (*n* = 3).

To explore the distribution of TTX in the liver, kidneys, small intestine, and blood, samples from these organs and blood were collected at different time points (2 h, 4 h, 6 h, 12 h, 24 h, 48 h, 72 h, and 168 h) after a single oral gavage of TTX in mice. The HPLC-MS/MS method was employed to determine the concentration of TTX (Figure 1). The liver and the kidney are important metabolic organs in organisms. They are the main target areas for metabolizing toxic substances [21]. The small intestine is an essential organ for digestion and absorption [22]. TTX introduced into the digestive tract of mice was transferred to the body and subsequently excreted over time. The tetrodotoxin concentration in mouse liver reached its highest concentration (72.67 ng/g, Figure 1) after 2 h of ingestion. A time-dependent decrease in tetrodotoxin concentrations was observed in the liver, kidney, and small intestine. In mouse blood, the tetrodotoxin concentration showed an increasing trend in the first hour and then decreased, indicating that, in mice, TTX is quickly distributed to the blood after ingestion. After 168 h, the tetrodotoxin concentration in mouse liver was 2.30 ng/g (Figure 1). At 72 h, tetrodotoxin concentration in mouse kidney was 2.64 ng/g (Figure 1). This may indicate a low capacity for liver and kidney accumulation of tetrodotoxin in mice. Over time, the tetrodotoxin concentration in mice gradually decreased until its elimination was almost complete. The elimination cycle of tetrodotoxin in mice is about 168 h (7 days).

### 2.2. Toxicity of TTX after Daily Oral Administration for 7 Days

#### 2.2.1. TTX Lethality and Distribution in Selected Tissues and Feces

Considering that the single oral dose of TTX (75 μg/kg) was almost completely eliminated from the liver, kidneys, blood, and small intestine of mice within 7 days, the toxin distribution in the selected tissues and feces was evaluated also after daily oral administration of TTX for 7 days, at three dose levels (25, 75 and 125 μg/kg). During the experiment, all mice in the low-dose (25 μg/kg TTX) and middle-dose (75 μg/kg TTX) groups were alive and showed no signs of behavioral changes or toxicity. However, two mice gavaged with high-dose (125 μg/kg TTX) died after 1 h of gavage on the third and sixth day, respectively, which is similar to the results of repeated exposure to chronic experiments at this dose [23].

The TTX distribution in selected tissues and feces after 7 days of daily oral administration are shown in Table 1. The concentration of TTX in mouse liver, kidney, blood, small intestine, and feces increased with increasing doses of TTX. The TTX concentration in feces was much higher than that in other tissues. The TTX concentration in mouse tissues was highest in the small intestine, second in the liver, third in blood, and lowest in the kidney.

#### 2.2.2. Effects of Different Doses of TTX on Oxidative Stress Markers in Mouse Liver

**Figure 2:**
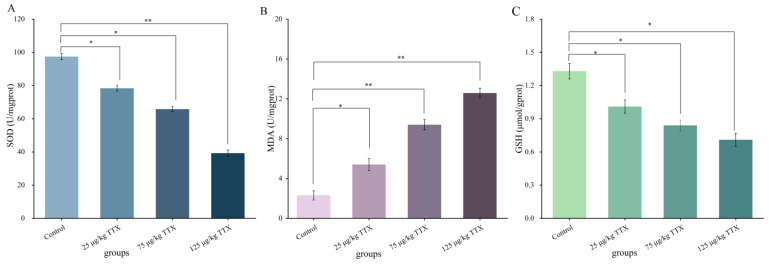
Effects of TTX on markers of oxidative stress in mouse liver. The oxidative stress indicators were SOD (**A**), MDA (**B**), GSH (**C**). Data represent mean ± SE (*n* = 5). * *p* < 0.05, ** *p* < 0.01.

MDA content is a significant marker of the body’s antioxidant capability, reflecting the speed and strength of peroxide oxidation and the indirect extent of peroxidative damage to tissues [24]. GSH is one of the indexes of the antioxidant capacity of organisms [25]. SOD is the most important free radical scavenger, which maintains the metabolic balance of the organism [26]. The effects of different doses of TTX on oxidative stress markers in mouse liver are shown in Figure 2. The content of MDA in mice of the high-dose group (125 μg/kg TTX) reached its highest value (12.65 U/mgprot, Figure 2B), which was a twofold increase over the control group. On the contrary, the content of SOD and GSH in mice of the high-dose group (125 μg/kg TTX) was the lowest (43.40 U/mgprot and 0.12 µmol/gprot, Figure 2A,C, respectively), which decreased to 47.6% and 87.8%, respectively, compared with the control group. The content of MDA in the liver increased significantly with the increasing dose of TTX, compared with the control group (*p* < 0.05). In the meantime, the content of SOD and GSH in the liver decreased significantly (*p* < 0.05). These results suggest that TTX causes the antioxidation state of disorder in mouse liver and the liver function is damaged.

#### 2.2.3. Effects of Different Doses of TTX on Oxidative Stress Markers in Mouse Kidney

**Figure 3:**
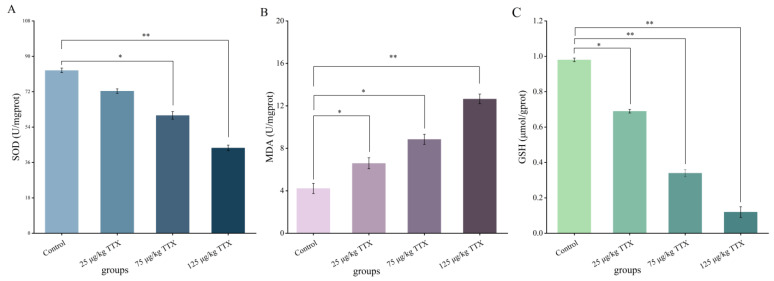
Effects of TTX on markers of oxidative stress in mouse kidney. The oxidative stress indicators were SOD (**A**), MDA (**B**), GSH (**C**). Data represent mean ± SE (*n* = 5). * *p* < 0.05, ** *p* < 0.01.

Studies have shown that oxidative damage is caused by the excessive production of lipid peroxide molecules and protein oxidation products, decreasing the activity of antioxidant enzymes [27]. The effects of TTX on oxidative stress markers in mouse kidney are shown in Figure 3. The results were similar to those obtained in the liver. The content of MDA in mouse kidney of the high-dose group (125 μg/kg TTX) reached its highest value (12.58 U/mgprot, Figure 3B), which far exceeded that of the control group (2.31 U/mgprot). The content of SOD and GSH in mice of the high-dose group (125 μg/kg TTX) was the lowest (39.26 U/mgprot and 0.71 µmol/gprot, Figure 3A,C, respectively). Compared to the control group, the levels of SOD and GSH decreased by 59.7% and 46.6%, respectively. The content of SOD and GSH in the kidney decreased significantly with the increasing doses of TTX after 7 days of continuous gavage, compared with the control group (*p* < 0.05). This result may indicate that the mouse kidney was damaged and its antioxidant capacity was decreased. The MDA level increased considerably with increasing doses of TTX (*p* < 0.05), which may indicate that the damage degree of renal peroxidation increased and renal function was damaged.

#### 2.2.4. Effects of Different Doses of TTX on the Liver and Kidney Function Markers in Mouse Blood

**Figure 4:**
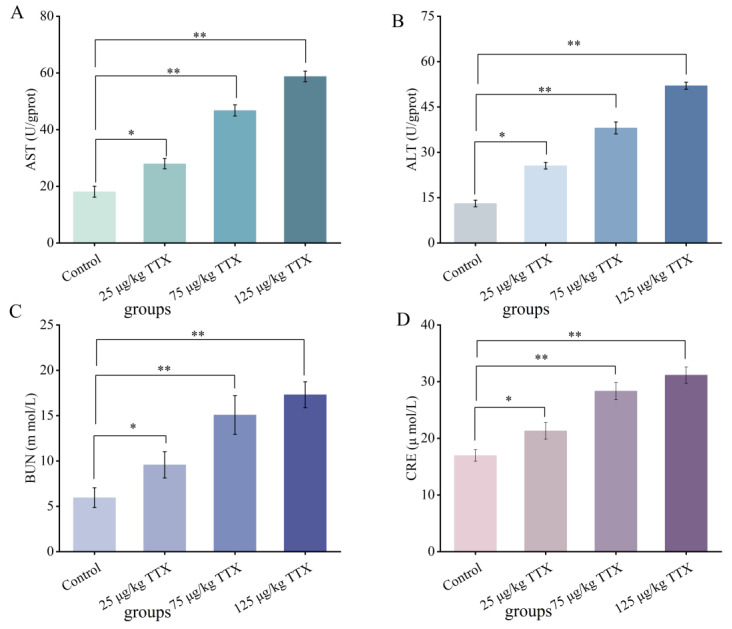
Effects of TTX on markers of the liver and kidney function in mouse blood. The oxidative stress indicators were AST (**A**), ALT (**B**), BUN (**C**) and CRE (**D**). Data represent mean ± SE (*n* = 5). * *p* < 0.05, ** *p* < 0.01.

AST and ALT are sensitive indicators that reflect liver damage [27]. BUN and CRE are markers of kidney damage [28]. Elevated AST and ALT levels indicate liver function damage, while elevated BUN and CRE levels indicate renal function damage. The effects of different doses of TTX on serum markers of liver and kidney function are shown in Figure 4. The activity levels of AST (Figure 4A), ALT (Figure 4B), BUN (Figure 4C), and CRE (Figure 4D) were consistently (*p* < 0.05) reduced in all experimental groups with increasing doses of TTX compared to the control group. These results suggest that TTX caused damage to the liver and kidney functions of mice. The damage degree of liver and kidney function increased with the increasing doses of TTX.

#### 2.2.5. Histopathological Effects of TTX on Mice’s Liver and Kidney

**Figure 5:**
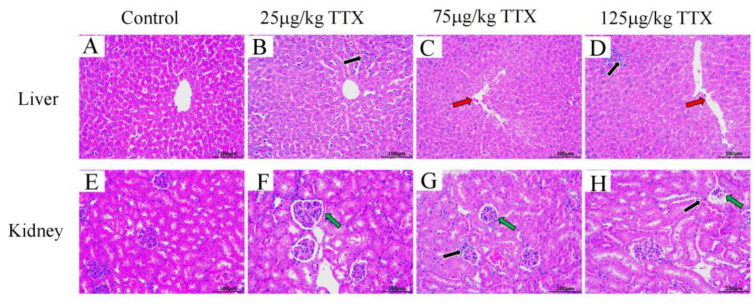
Changes due to effects of TTX on mouse liver and kidney. (**A**,**E**) show mice’s liver and kidney tissue of the control groups, respectively. (**B**–**D**) show the liver tissue of the low-dose (25 μg/kg TTX), middle-dose (75 μg/kg TTX), and high-dose (125 μg/kg TTX) groups, respectively. (**F**–**H**) show the kidney tissue of the low-dose (25 μg/kg TTX), middle-dose (75 μg/kg TTX), and high-dose (125 μg/kg TTX) groups, respectively. The black arrow shows inflammatory cell infiltration, the red arrow shows irregular hepatic sinuses, and the green arrow shows renal tubular changes. H&E stained, 200× magnification.

The liver and kidney were selected for histopathological analysis because they are the main organs affected by the metabolic response to the toxicant. Histopathological manifestations in mouse liver and kidney are shown in Figure 5. Compared with the control group (Figure 5A), all experimental groups showed hepatocellular steatosis and inflammatory cell infiltration in the liver tissue (Figure 5B–D). The degree of inflammatory cell infiltration extended with increasing doses of TTX. Mild dilatation of the hepatic sinusoids was observed in the low-dose group (25 μg/kg TTX, Figure 5B). In contrast, in the middle-dose group (75 μg/kg TTX, Figure 5C) and high-dose group (125 μg/kg TTX, Figure 5D), irregularity of the hepatic sinusoids was evident, and the hepatic cords also became increasingly disorganized with increasing doses of TTX. Compared with the control group (Figure 5E), inflammatory cell infiltration and vacuolization were detected within the renal tissue of all experimental groups (Figure 5F–H). The degree of cell infiltration and vacuolization was intensified with increasing doses of TTX. Enlarged renal tubular swelling was also observed in the low-dose group (25 μg/kg TTX, Figure 5F). In the middle-dose (75 μg/kg TTX, Figure 5G) and high-dose (125 μg/kg TTX, Figure 5H) groups, the renal cell arrangement disorder was prominent and the degree of disorder was positively correlated with the dose of TTX.

## 3. Discussion

The frequent detection of TTX in different edible marine organisms from wide geographical areas poses an increasing risk for consumers’ health. In this study, the results of the single-dose administration experiment show that the elimination cycle of a single dose of 75 μg/kg TTX in mice is about 168 h (7 days). In the single-dose administration experiment, TTX was quickly distributed to all body parts of mice 2 h after it was gavaged. At this time, the content of TTX in different mouse tissues was liver > small intestine > blood > kidney (Figure 1). It has been found that TTX is rapidly absorbed by rats after intramuscular injection; in the first 10 min it is found in plasma, mainly distributed in stomach, kidney, and intestine [29]. Furthermore, at the 0.5 h mark, the level of TTX was greater in the rat’s stomach, lung, kidney, and heart compared to that in the blood. Matsumoto et al. discovered that the administration of tetrodotoxin into the digestive system of non-toxic pufferfish was followed by the detection of tetrodotoxin in the blood within 30 min [30]. Abal et al. reports that oral administration of TTX leads to ultra-microstructural cytological damage in the liver, spleen, and intestine of mice within 2 h [29]. This indicates that the tissue distribution of TTX in mice after ingestion is different from that of other animals and is influenced by the mode of administration [31,32].

Comparing Figure 1 and Table 1, the TTX concentration in the liver, kidney, and blood at 24 h after one-time and continuous gavage appears very similar. This may imply that mice have a lower accumulation capacity. The levels of the oxidative stress markers SOD and GSH in both liver and kidney significantly decreased with increasing TTX doses. In contrast, MDA levels significantly increased with increasing TTX doses in the daily oral administration experiment. The ability of a low dose of TTX to affect the oxidative state of the liver and kidney may demonstrate the role of oxidative stress in this mechanism of toxicity. Currently, the mechanism of acute toxicity of TTX is usually reported to be a high degree of blockage of sodium channels on nerve cell membranes, blocking the conduction of nerve impulses and causing paralysis of central and peripheral nerves, leading to a toxic response [33,34]. The present study focused on the destructive ability of tetrodotoxin on the liver and kidney of mice. The effects of low doses of tetrodotoxin on degenerative processes in the nervous system of mice will be the subject of further studies.

The results presented in Figure 2 and Figure 3 showed that the MDA content in the liver and kidney significantly increased; the SOD activity decreased significantly in the liver (47.6%) and kidney (59.7%); and the GSH content also decreased significantly in both organs of TTX-treated mice. This decrease was more evident in the kidney than in the liver. Moreover, the present results showed that TTX induced an increase in the liver damage indicators, AST and ALT [27], as well as in the markers of kidney damage, BUN and CRE [28], in the blood in all doses of TTX-treated mice (Figure 4). Finally, the histopathological analysis demonstrated that, in all the TTX-treated mice, the liver and kidney cells were damaged to varying degrees (Figure 5). These results show that mice have hepatotoxicity and nephrotoxicity after daily oral administration of TTX for 7 days, and the hepatotoxicity is more prominent than the nephrotoxicity. There is still very limited information on organ damage caused by ingestion of a low dose of tetrodotoxin. It has been shown that the effects of continuous oral administration of TTX (125 μg/kg, for 28 days) in mice are mainly cardiac and renal damage, with less effect on the liver [23]. This effect is different from the findings of the present study and can be influenced by the duration of the gavage and the strain of the mice. Abal et al. [29] observed significant ultrastructural changes (vacuolization of cells, swelling of mitochondria and disruption of cell membranes, etc.) in the liver, spleen, and intestines of experimental animals after 2 h of oral administration of TTX, suggesting that oral administration of TTX can lead to cellular damage in these organs within a short period of time. Thus, TTX damage to organ cells does need to be taken seriously, and provides a reference for the effects of accidental ingestion of a low dose of TTX in humans.

The present study has certain limitations due to its focus on the oral administration of pufferfish toxin for 7 days and the measurement of toxicity in specific tissues (liver, kidney, and small intestine) of mice, which may restrict the comprehensive evaluation of TTX toxicity. However, future research should encompass a broader range of tissues and fluids to provide a more comprehensive assessment of TTX toxicity. For instance, investigations into the effects and potential toxicities of TTX in tissues such as skin, lungs, heart, and urine can shed light on its actions in different organs. In addition, exploring the metabolism and excretion of TTX will further enhance our understanding of its mechanism of action, toxic effects, and potential health risks. Furthermore, extrapolation of findings from animal models to human populations must be considered as a limitation, and future studies should explore the effects of TTX in relevant human cell lines or in vitro models to bridge the gap between animal and human studies.

## 4. Conclusions

In this study, the elimination cycle of a single oral dose of TTX (75 µg/kg) in mice was approximately 168 h (7 days). The dose-dependent toxic effects of TTX on the liver and kidneys of mice were found by comparing the toxic effects of daily oral administration of 25, 75, and 125 µg/kg TTX for 7 consecutive days. Histopathological examination revealed that inflammatory cell infiltration in the liver and kidney became severe with increasing doses of TTX. It was also observed that the hepatic cord also became disorganized and the renal tubular cell arrangement was significantly disorganized (75 µg/kg TTX and 125 µg/kg TTX). TTX was also found to be more hepatotoxic than nephrotoxic in mice. It is hoped that these results will inform the study of accidental ingestion of low doses of TTX in humans and draw attention to food safety. In summary, while the current study provides valuable insights into the liver and kidney toxicity of TTX in mice, future research should aim to broaden the scope by investigating additional tissues and fluids. This will enable a more comprehensive assessment of TTX toxicity, unravel its mechanisms of action, and better understand the potential health risks it poses. Additionally, exploring human cell lines and conducting epidemiological studies will enhance our understanding of the translational implications of these findings.

## 5. Materials and Methods

### 5.1. Chemicals and Reagents

TTX standard (CAS Number: 4368-28-9, Molecular Formula: C_11_H_17_N_3_O_8_), with a purity of 98%, was purchased from Dr. Ehrenstorfer GmbH, Augsburg, Germany. Chromatographic-grade formic acid, acetic acid, methanol, acetonitrile, and ammonium acetate were obtained from Sigma-Aldrich Co. (Saint Louis, MO, USA). Analytical-grade solvents including Na_2_HPO_4_, NaH_2_PO_4_, NaCl, and NaOH were obtained from the China National Pharmaceutical Group Corporation (Sinopharm, Shenzhen, China). All other reagents utilized in the research were of analytical grade.

### 5.2. Animals

SPF-grade ICR male mice (6 weeks old, weighing 20 ± 2 g) were purchased from the Zhejiang Academy of Medical Sciences. The animal production license was SCXK (Zhejiang) 2014-0001. They were kept in the animal laboratory of the Zhejiang Institute of Marine Fisheries and were given a standard diet and water under controlled conditions (room temperature 23 ± 1 °C, light and dark cycle at 12:12, relative humidity 40–60%). They were kept adaptively for one week before the experiment.

### 5.3. Single-Dose Administration

According to the NOAEL value of oral TTX in mice [13], mice were oral gavaged (75 µg/kg TTX) after fasting without water for 12 h before the oral administration. The mice were divided into 9 groups (*n* = 3). Mice in the control group received the same volume of 0.1% acetic acid aqueous solution. Samples (blood, liver, kidney, and small intestine) were taken at 2 h, 4 h, 6 h, 12 h, 24 h, 48 h, 72 h, and 168 h after gavage. Blood samples were collected at the defined time periods from the control and dose groups for serum biochemistry analysis as mentioned above. Blood was drawn from the orbital sinus. Serum fractions were promptly isolated by centrifugation at 4000 rpm for 10 min and kept at −80 °C until analysis. Mice were then sacrificed in a CO_2_ chamber at the specified time points. The liver, kidney, and small intestine of the mice were removed with normal saline at 4 °C and stored at −80 °C for TTX detection.

### 5.4. Daily Oral Administration for 7 Days

Thirty-two mice were randomized into four groups of eight mice each: control, low-dose, medium-dose, and high-dose. The mice were orally gavaged once daily for 7 days with TTX (dose groups: 25 µg/kg, 75 µg/kg, and 125 µg/kg BW, solubilized in a 0.1% aqueous solution of acetic acid). The concentration of TTX in each dose group was set according to the LD_50_ value of TTX taken orally by mice [13,23]. The control group was given the same volume of 0.1% acetic acid aqueous solution. Twenty-four hours after the last dose, animals were sacrificed in a CO_2_ chamber and blood was collected by the eyeball extraction method with normal saline at 4 °C for TTX detection. The liver and kidneys of the mice were removed for biochemical analysis. Small pieces of liver and kidney were removed to perform histological sections for pathological examination. The liver, kidney, and small intestine of the mice were removed with normal saline at 4 °C and stored at −80 °C for the determination of tissue biochemical indexes and TTX content in tissues. On the last day, the feces in each dose group were collected for TTX content determination.

### 5.5. HPLC-MS/MS Analysis

#### 5.5.1. TTX Extraction

An amount of 2 g of the liver, kidney, small intestine, and feces, and 1 mL of blood, were homogenized. The samples were placed in a 50 mL centrifuge tube and 10 mL of 1% acetic acid and methanol solution was added and mixed by vortexing for 6 min, and then exposed to ultrasonic treatment for 15 min at 60 °C. After returning to normal temperatures, the samples were spun at 7000 rpm for 5 min. Twenty-five mL of phosphate buffer saline (PBS) was added to the 5 mL supernatants. The samples were then shaken, had the pH adjusted to neutral (6.9~7.2) with an appropriate amount of sodium hydroxide solution (1 mol/L), and purified. A TTX immunoaffinity column (Jiangsu Meizheng Biotechnology Co., Ltd., Wuxi, China) was fixed, which had been returned to room temperature on the solid-phase extraction device. The protective solution was first drained, the sample solution was added, and the flow rate was kept at one drop/s. When the sample solution was completely purified, it was eluted with 20% methanol aqueous solution (8 mL) and then the liquid in the TTX immunoaffinity column was air dried. Finally, 2% acetic acid methanol solution (4 mL) was used for elution, and the eluent was collected. The eluent was dried with nitrogen at 45 °C, dissolved with 1 mL of 0.2 mmol/L aqueous sodium acetate/acetonitrile (*v*/*v* = 1/9) solution with 0.1% aqueous acetic acid, mixed by vortexing for 30 s, filtered with 0.22 µm organic phase filter membrane into the injection bottle, whereupon the TTX content was detected.

#### 5.5.2. HPLC-MS/MS Analysis of TTX

TTX was measured on an ACQUITY ultra-performance liquid chromatography tandem triple quadrupole mass spectrometer (Waters Co., Milford, MA, USA). TTX was identified and quantified according to the method previously described [35]. The toxin was separated using an ACQUITY UPLC BEH Amide column (50 mm × 2.1 mm, 1.7 µm, Waters Co., Milford, MA, USA) at 40 °C with a sample volume of 10 µL. The initial mobile phase A was composed of 5 mmol/L aqueous ammonium acetate with 0.1% formic acid, and the initial mobile phase B was acetonitrile (A:B = 1:9). The elution was gradient elution (0–0.50 min, 10% A, 90% B; 1.50–4 min, 60% A, 40% B; 4.50–5 min, 10% A, 90% B) with a flow rate of 0.30 mL/min.

The electrospray ion source was used in positive ion mode (EMS+) and analyzed in Multiple Reaction Monitoring (MRM) mode with the following settings: TTX *m*/*z* 320→*m*/*z* 302; capillary voltage 3.50 kV; desolvation gas temperature 385 °C; ion source temperature 119 °C; cone gas orifice, high purity nitrogen; flow rate 55 L/h; desolvation gas flow rate 800 L/h; cone hole voltage 30 V; and collision energy 25 V.

### 5.6. Liver and Kidney Function Markers

Glutathione (GSH), malondialdehyde (MDA), and superoxide dismutase (SOD) activity were determined using commercial kits based on instructions supplied by the manufacturer (Nanjing Jiancheng Biological Co., Ltd., Nanjing, China).

### 5.7. Serum Biochemical Indexes

Glutathione (ALT), glutathione aminotransferase (AST), urea nitrogen (BUN), and creatinine (CRE) were determined using commercial kits based on instructions supplied by the manufacturer (Nanjing Jiancheng Biological Co., Ltd., Nanjing, China).

### 5.8. Histological Analysis

The liver and kidney mouse tissues were washed with saline, fixed in 4% paraformaldehyde solution for 24 h, deparaffinized, and processed for paraffin embedding. Each sample was sliced into sections, stained with hematoxylin–eosin (H&E), and finally sealed. The histopathological changes of the liver and kidney were observed under the light microscope (Olympus BX51 microscope, Olympus Inc., Tokyo, Japan) and photographed.

### 5.9. Data Analysis

The experimental results were statistically analyzed using SPPS 26.0 (IBM Co., Armonk, NY, USA). Pearson’s coefficient was chosen for correlation analysis and Duncan’s test was used for multiple comparisons for one-way analysis of variance (ANOVA). The data were represented as mean ± standard deviation. A *p*-value < 0.05 was considered to be statistically significant between the dose groups and the control group (* *p* < 0.05, ** *p* < 0.01).

## Figures and Tables

**Table 1:** TTX distribution in selected tissues and feces. Data represent mean ± SE (*n* = 3).

Group	Liver (ng/g)	Kidney (ng/g)	Blood (ng/g)	Small Intestine (ng/g)	Feces (ng/g)
25 μg/kg TTX	34 ± 2	14 ± 3	27 ± 2	34 ± 2	50 ± 3
75 μg/kg TTX	41 ± 1	16 ± 2	32 ± 2	57 ± 3	99 ± 3
125 μg/kg TTX	46 ± 2	20 ± 2	42 ± 2	86 ± 2	150 ± 3

## Data Availability

The data presented in this study are available on request from the corresponding author. The data are not publicly available due to privacy or ethical restrictions.
